# Correlation analysis between occlusal plane and maxillofacial alterations in Chinese patients with an anterior disc displacement

**DOI:** 10.1186/s12903-025-07031-w

**Published:** 2025-10-21

**Authors:** Binjie Hou, Wanfang Xiang, Xiaohan He, Yajie Fu, Xin Yu, Xiaojing Pan, Min Wang

**Affiliations:** 1 School/Hospital of Stomatology, Lanzhou University, 199, Donggang West Road, Chengguan District, Lanzhou, Gansu Province 730000 PR China; 2 School of Basic Medical Sciences, Lanzhou University, No.199, Donggang West Road, Chengguan District, Lanzhou, Gansu Province 730000 PR China

**Keywords:** Temporomandibular joint (TMJ), Anterior disc displacement(ADD), Occlusal plane, Craniomaxillofacial morphology measurement

## Abstract

**Objective:**

To study the changes in the vertical and sagittal craniomaxillofacial morphology in Chinese adult patients with bilateral anterior temporomandibular joint(TMJ)disc displacement and to explore their correlation. This may provide valuable insights for clinical diagnosis and treatment planning.

**Methods:**

Ninety-eight Chinese adult patients were divided into 3 groups: 29 patients in bilateral disc Normal Position group (BN), 33 patients in bilateral Anterior Disc Displacement With Reduction group (ADDWR) and 36 patients in bilateral Anterior Disc Displacement Without Reduction group (ADDWoR). Uceph software was used to measure 6 items of occlusal plane and 13 items of craniomaxillofacial morphology for comparison and correlation analysis between groups.

**Results:**

Compared to the BN group, subjects with anterior TMJ disc displacement (ADD) were more likely to exhibit a steeper posterior occlusal plane and an extended craniofacial posture with a Class II hyperdivergent pattern characteristics. Nevertheless, there were no statistically significant differences between the ADDWR and ADDWoR groups. The results of Pearson correlation analysis revealed a significant association between a steep occlusal plane in ADD patients and both skeletal mandibular retraction and vertical overgrowth.

**Conclusion:**

Chinese patients with anterior disc displacement of the TMJ have a steeper posterior occlusal plane and hyperdivergent skeletal Class II malocclusions tendency, which may be associated with occlusal function or condylar remodeling.

Temporomandibular disorders (TMD) refer to a group of diseases that involve the temporomandibular joint and/or masticatory muscle system characterized by associated clinical symptoms such as pain, snapping, aberrant movement of the mandible, etc. The incidence of TMD is relatively high and varies among different groups. The occurrence of the disease reaches its highest point between the ages of 15 and 19, and then stabilizes beyond the age of 20 [1]. The etiology and mechanisms of TMD are complex and multifactorial, including malocclusion, occlusion, muscular problems, trauma, parafunctional activities, deep somatic pain, hormone, hereditary and psychosocial factors [2]. And Increased discrepancy between the CR (centric relation) and MI (maximum intercuspation) aggravates the orthopedic instability and usually leads to faster development of intracapsular and extracapsular disorders [3, 4]. Internal disorders (ID) of the TMJ are a common cause of TMD symptoms, with anterior disc displacement (ADD) being the most prevalent form. Several studies have demonstrated that ADD is the primary structural disorder that causes symptoms of TMD [5]. ADD can be further categorized into two types: anterior disc displacement with reduction (ADDWR) and anterior disc displacement without reduction (ADDWoR).

The occlusal plane (OP), as defined by Downs, is an imaginary plane that has a substantial impact on the craniomaxillofacial framework [6]. However, in patients with a pronounced curve of Spee, the inclinations of the anterior and posterior teeth may differ. Consequently, the anterior occlusal plane (AOP) and posterior occlusal plane (POP) are used to determine the inclinations of the anterior and posterior teeth, respectively [7]. Evidence suggests that the OP influences factors such as the height of the mandibular ramus [8]. In the sagittal dimension, Kim [9–11] identified the occlusal plane as a primary factor influencing sagittal craniomaxillofacial morphology. Mandibular retraction and clockwise rotation are correlated with a steep posterior occlusal plane within the vertical dimension [12].

Previous research has established a significant association between ADD and changes in craniomaxillofacial morphology, including deep overbite, open bite, diminished posterior facial height, lowered mandibular ramus height, and clockwise rotation of the mandible [13–16]. Nevertheless, there is a lack of much study in Chinese populations on the correlation between ADD, occlusal plane, and craniomaxillofacial morphological indicators. Hence, the objective of this study is to compare craniofacial morphological measurement indicators, such as OP, between patients with anterior disc displacement and normal individuals using lateral cephalometric imaging, and examine the relationship between the inclination of the occlusal plane and craniomaxillofacial morphological indicators in ADD patients in comparison to normal subjects. This will enable doctors to evaluate the craniomaxillofacial growth patterns of ADD patients and forecast the likelihood of ADD by employing cephalometric radiometric imaging.

## Materials and methods

According to the position of temporomandibular joint disc, the status of temporomandibular joint disc can be divided into 3 types [13].

1. Bilateral disc normal position. In the closed-mouth position, the intermediate zone of the disc was interposed between the condyle and the posterior slope of the articular eminence, with the anterior and posterior bands equally spaced on either side of the condylar load point.
2. Anterior disc displacement with reduction. The TMJ disc moves forward relative to the normal anatomical position, but when the mandible moves (such as opening the mouth or extending forward), the disc can be temporarily reset to the normal position, accompanied by characteristic snapping or rubbing sound.
3. Anterior disc displacement without reduction. The position of joint disc was anteriorly displaced relative to the posterior slope of the articular eminence and the head of the condyle, and it cannot reset itself during the opening and closing movement of the mandibular condyle, hindering the sliding of the condyle.

### Study subjects

**Fig. 1:**
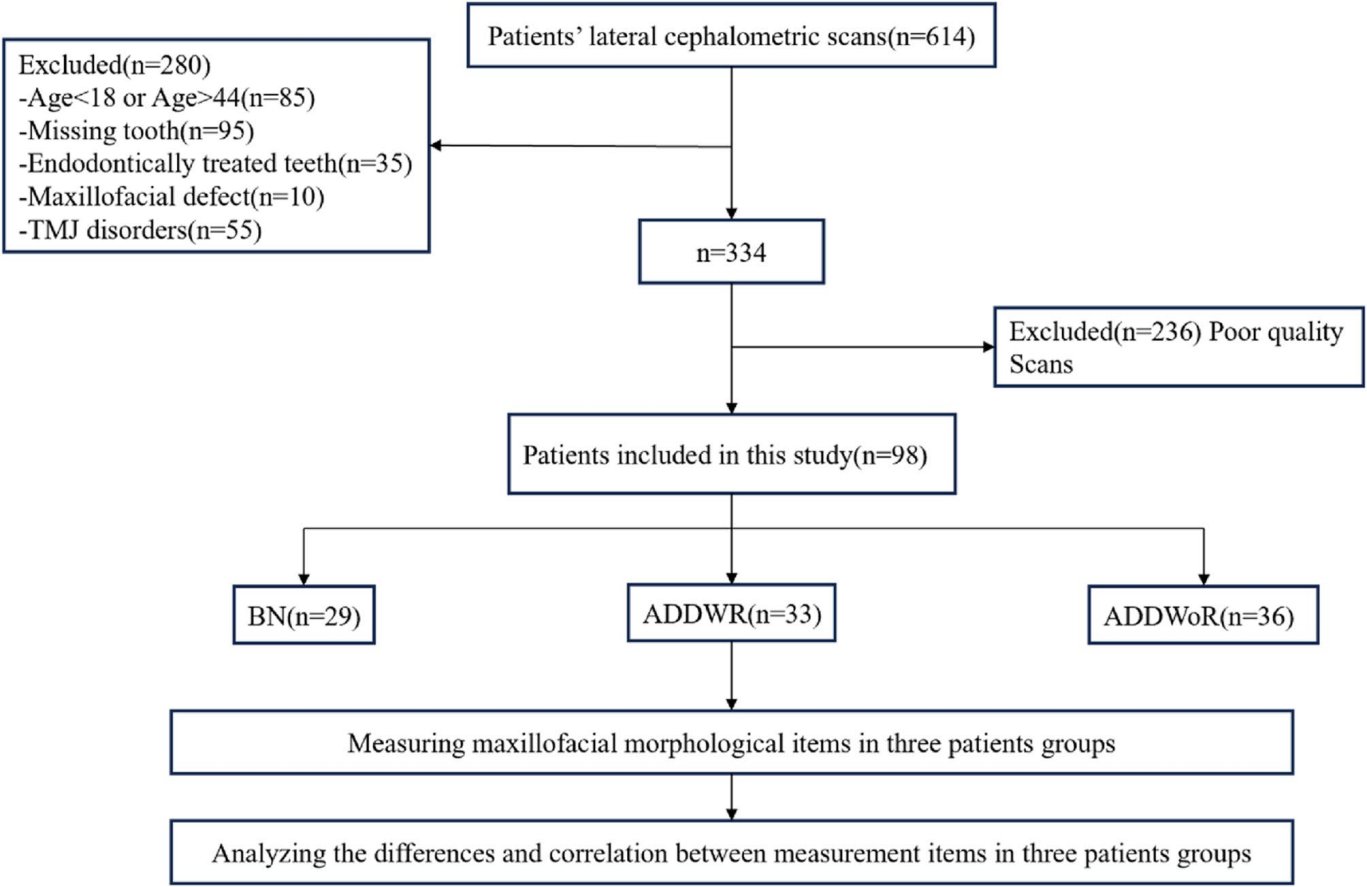
Experimental procedure flowchart (Inclusion and exclusion criteria refer to Supplementary Table S1)

We design and implement a cross-sectional retrospective study to reasonably address the research objectives. Patients diagnosed with ADDWR or ADDWoR and admitted to the Temporomandibular Joint Specialist Clinic, Lanzhou University Stomatology Hospital, China, from January 2020 to December 2024 constituted the study samples (Fig. 1). The subjects were diagnosed as bilateral ADD or BN by two experienced joint specialists concurrently. The diagnosis was strictly based on RDC/TMD(Research Diagnostic Criteria for Temporomandibular Disorder)about symptom questionnaires and clinical examinations that consider medical history and oral and maxillofacial function examinations. The study protocol was approved by the Ethics Committee for Clinical Scientific Research of Lanzhou University School of Stomatology.

### Investigational equipment

All subjects underwent cephalometric lateral radiograph examination in the Department of Radiology, Stomatology Hospital of Lanzhou University. The equipment used was ORTHOPHOSSL3D (Sirona Dental System GmbH, Germany). During imaging, all subjects were instructed to sit straight, keep Frankfort horizontal plane parallel to the ground, maintain the intercuspal position (ICP), refrain from swallowing and hold their breath till the imaging process was complete. This ensured that all participants were photographed in a consistent natural head position.

### Measurement process

In this study, to ensure the accuracy and reliability of the measurements, all tests were conducted by a rigorously calibrated tester, who had no knowledge of the presence or absence of ADD in the 98 patients selected for the study, identified the landmarks and reference planes on the cephalometric lateral radiograph of all the patients and measured them twice, one week apart, then take the average value as the data of this study.

### Measurement items

**Fig. 2:**
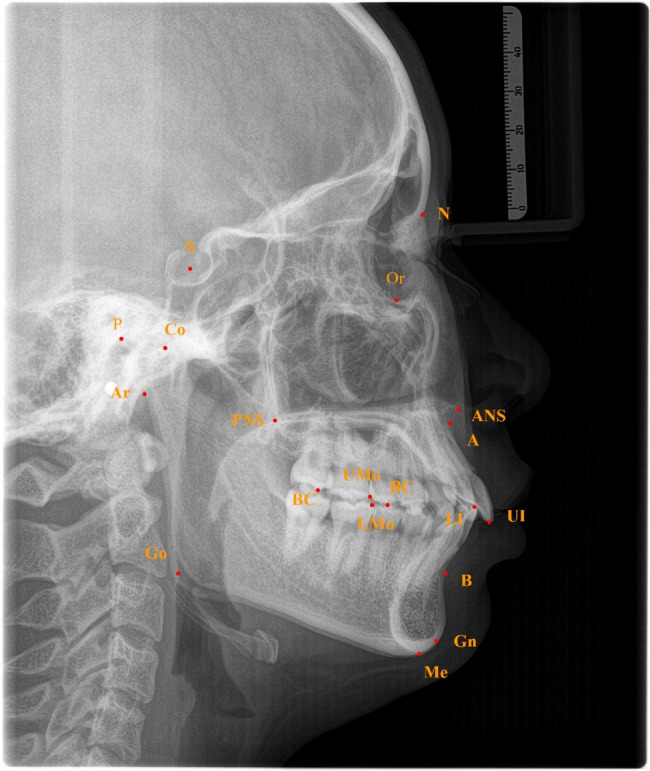
Landmarks used in this study:1.N(nasion); 2.S(sella); 3.Or(orbitale); 4.P(porion); 5.ANS(anterior nasal spine); 6.PNS(posterior nasal spine); 7.Ar(articulare); 8.Point A; 9.Point B; 10.Me(menton); 11.Go(gonion); 12.UI(upper incisor); 13.LI(Lower incisor); 14.BC(buccal cusp); 15.UMo(upper molar);16. LMo(lower molar); 17.Co(condylion);18.Gn(gnathion)

**Fig. 3:**
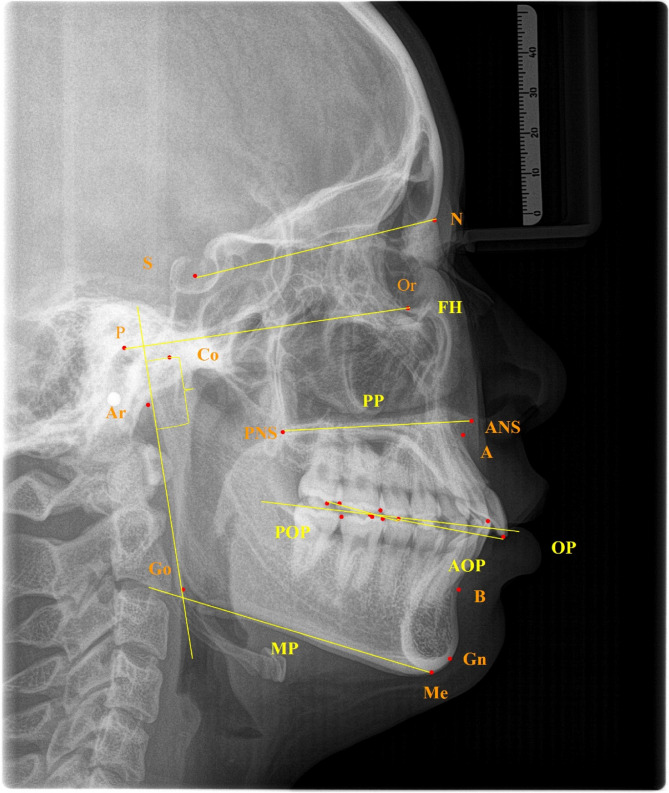
Craniosagittal reference planes used in this study:1. nasion-sella plane (SN, The plane through nasion and sella) 2. Frankfort horizontal plane (FH, The plane through porion and orbitale) 3. Palatal plane (PP, The Plane through the posterior nasal spine and anterior nasal spine) 4. Occlusal plane (OP, The line connecting the occlusal midpoint of the upper and lower first permanent molars with the midpoint of the upper and lower central incisors) 5. Anterior occlusal plane (AOP, The line connecting the upper incisor to the buccal cusp of the maxillary second premolars) 6. Posterior occlusal plane (POP, The line connecting the buccal cusp of the maxillary second premolar to the buccal cusp of the maxillary second molar) 7. Mandibular plane (MP, The line through gonion and menton)

Based on Uceph 4.2.1(Chengdu, Sichuan), we measured the following craniomaxillofacial morphological features. (Table 1; Figs. 2 and 3)

1. Occlusal measurement: OP-FH, AOP-FH, POP-FH, SN-OP, MP-OP, PP-OP
2. Craniomaxillofacial sagittal measurement: SNA, SNB, ANB
3. Craniomaxillofacial vertical measurement: FMA, MP-SN, Ar-Go-Me, PP-MP, N-S-Ar
4. Linear measurement: Ar-Go, Go-Me,Co-Gn, N-Me, S-Go, condylar height（mm）

**Table 1:** Craniofacial morphological measurements

	Measurement items	Definition
Occlusal feature	OP-FH	The angle between the occlusal plane (OP) and the Frankfort horizontal plane (FH)
	AOP-FH	The angle between the anterior occlusal plane (AOP) and the Frankfort horizontal plane (FH)
	POP-FH	The angle between the posterior occlusal plane (POP) and the Frankfort horizontal plane (FH)
	SN-OP	The angle between the nasion-sella plane (SN) and the occlusal plane
	MP-OP	The angle between the mandibular plane (MP) and the occlusal plane
	PP-OP	The angle between the palatal plane (PP) and the occlusal plane
Sagittal feature	SNA	The angle between SN and NA planes
	SNB	The angle between SN and NB planes
	ANB	The angle between AN and NB planes
Vertical feature	FMA	The angle between the Frankfort horizontal plane (FH) and the mandibular plane
	MP-SN	The angle between the nasion-sella plane (SN) and the mandibular plane
	Ar-Go-Me	The angle between the articulare-gonion plane and the mandibular plane
	PP-MP	The angle between the palatal plane and the mandibular plane
	N-S-Ar	The angle between the nasion-sella plane (SN) and the S-Ar lines
Linear measurement	Ar-Go	The distance from articulare (Ar) to gonion (Go)
	Go-Me	The distance from gonion (Go) to menton(Me)
	Co-Gn	The distance from condylion (Co) to gnathion (Gn)
	N-Me	The distance from nasion (N) to menton(Me)
	S-Go	The distance from sella (S) to gonion (Go)
	Condylar height	The distance between the lowest point of the sigmoid notch and the condylion on the plane of the mandibular ramus

### Statistical analysis

Statistical analysis was conducted using the Statistical Product and Service Solutions (SPSS) version 21.0 (IBM, USA). The Shapiro-Wilk test was used for normality testing, and the Levene test was employed to assess the homogeneity of variance. A two-sample independent t-test was used to compare the means between two groups. For variables not conforming to a normal distribution, the Kruskal-Wallis test was applied. For variables that did conform to a normal distribution, ANOVA was used if variances were homogeneous, while the Brown-Forsythe ANOVA was used if variances were heterogeneous. LSD-t tests were employed for each measurement to evaluate the average differences between sides for each element of the sample. When the data followed a normal distribution, the mean ± standard deviation (x ± S) description and Pearson correlation analysis were used. When the data did not follow a normal distribution, the median (P25, P75) description and Spearman correlation analysis were utilized. The absolute value of Pearson correlation coefficient between 0 ~ 0.2 indicates vary weak correlation, between 0.2 ~ 0.4 indicates weak correlation, between 0.4 ~ 0.6 indicates moderate correlation, and above 0.6 indicates strong correlation [17].

## Results

### Patients’ general characteristics

A total of 98 subjects were included in this study with an age range of 18 to 44 (mean age, 26.5 ± 6.7 years). There were no significant differences in age and sex distribution among the groups. (Table 2)

**Table 2:** Number and age distribution of subjects of BN, ADDWR and addwor groups

Group	BN	ADDWR	ADDWoR	Total	Significance
Subjects, n(%)	29(30.1%)	33(33.6%)	36(36.7%)	98(100%)	NS
Sex (M, F)	9,20	10,23	7,29	26,72	NS
Age(y)					
Mean	25.3 ± 6.0	25.5 ± 6.5	28.9 ± 7.5	26.5 ± 6.7	NS
Range	19–36	18–41	19–44	18–44	NS

*NS* Not significant

### Comparison of occlusal plane and craniomaxillofacial morphological characteristics between BN group and ADD group

**Fig. 4:**
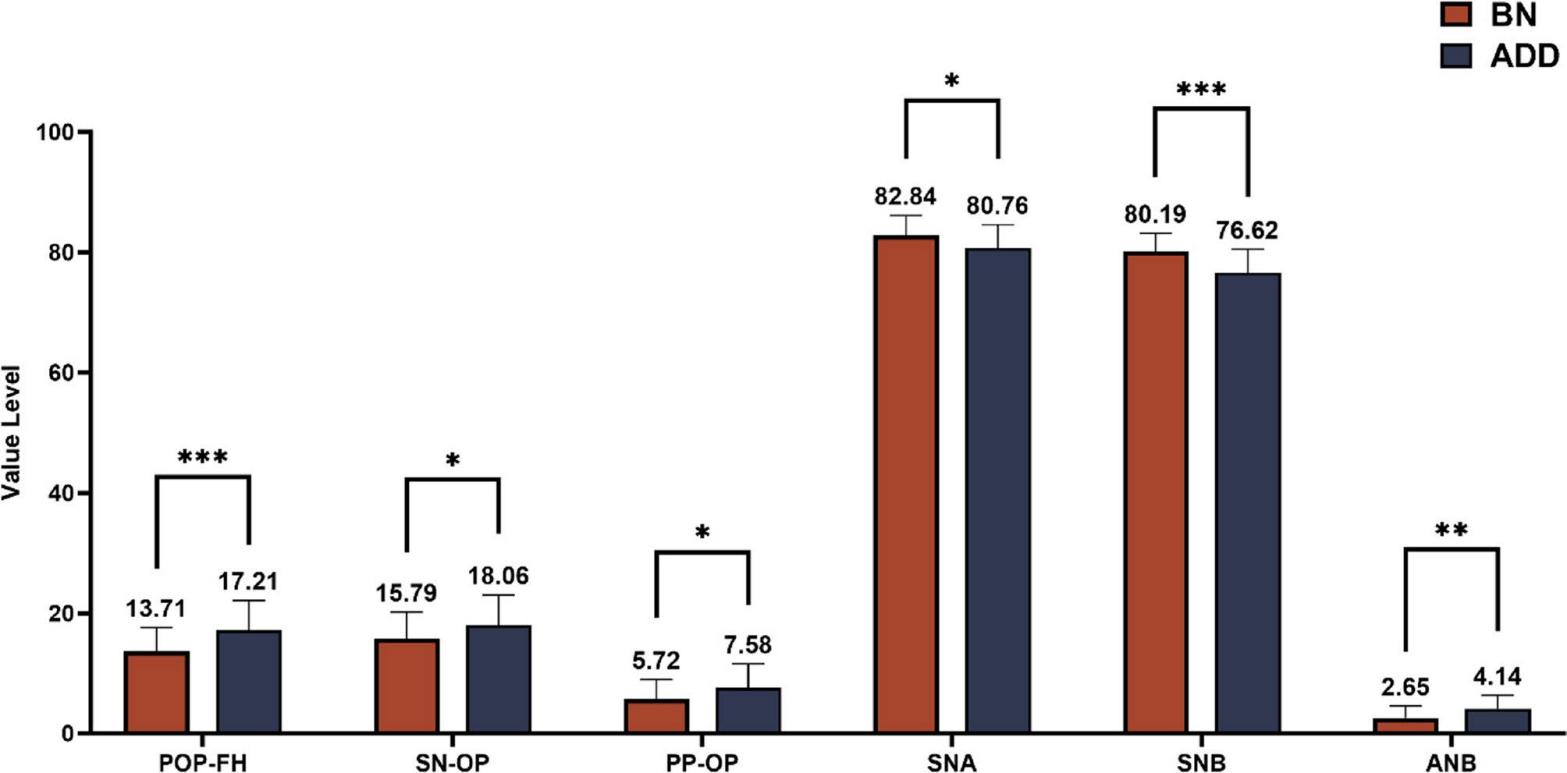
Significantly different measurements of craniomaxillofacial morphology between BN and ADD group. *p ≤ 0.05, **p ≤ 0.01, ***p ≤ 0.001 T-test is used to analyze data at the level of 0.05

**Fig. 5:**
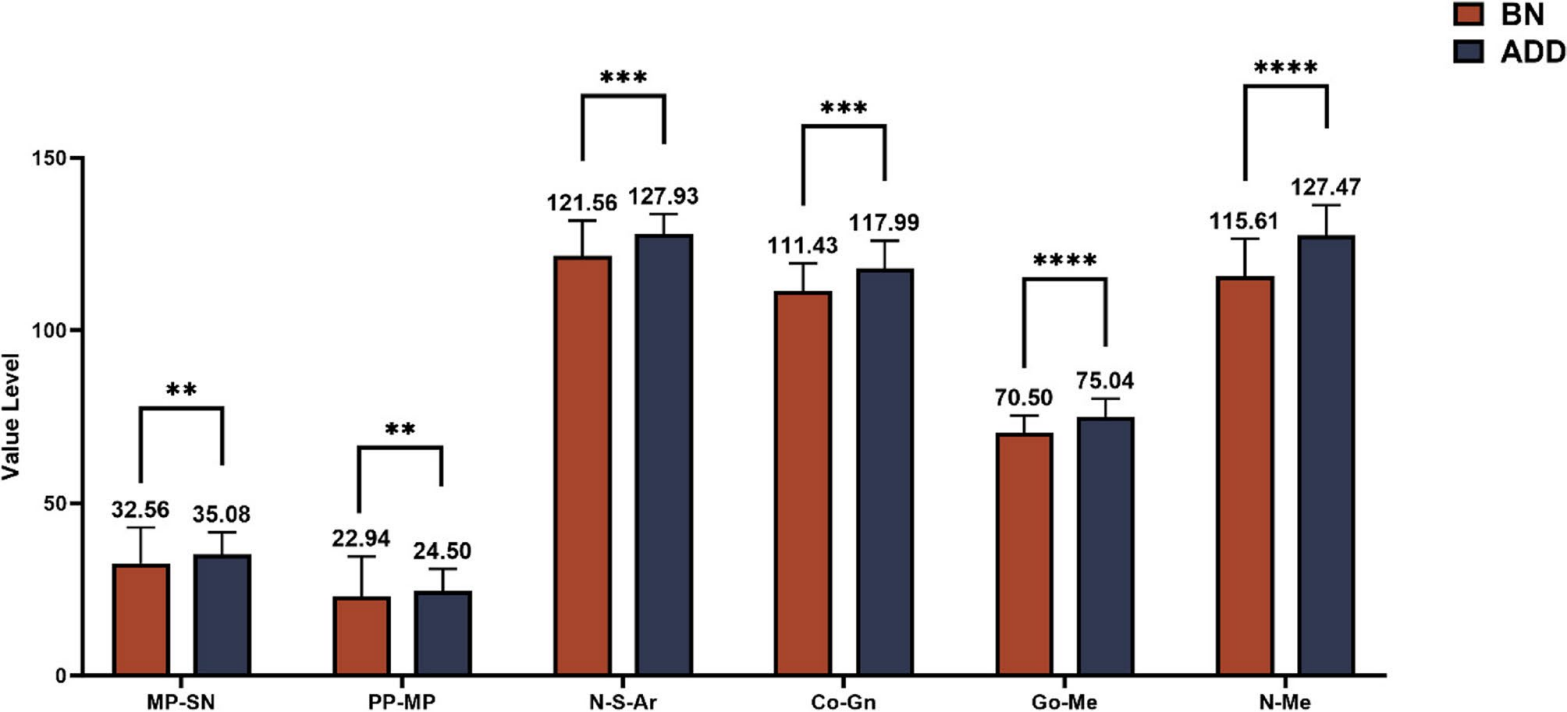
Significantly different measurements of craniomaxillofacial morphology between BN and ADD group. **p ≤ 0.01, ***p ≤ 0.001, ****p ≤ 0.0001 T-test is used to analyze data at the level of 0.05

Two independent sample t-tests was conducted between the BN and ADD groups, yielding the following results (Table 3; Figs. 4 and 5). The comparison of the occlusal plane and craniofacial morphological features between the BN and ADD groups is shown in the Table 3. The posterior occlusal plane (POP-FH, *P* < 0.001; SN-OP, *P* = 0.032; PP-OP, *P* = 0.03), sagittal indicators (SNA, *P* = 0.013; SNB, *P* < 0.001; ANB, *P* = 0.002), vertical indicators (MP-SN, *P* = 0.003; PP-MP, *P* = 0.004; N-S-Ar., *P* = 0.001), and linear indicators (Go-Me and N-Me, *P* < 0.001) exhibited statistically significant differences.

The results demonstrate that compared to BN group, ADD groups exhibitedan increase in the occlusal indicators, retraction of the maxilla and mandible, an increase in the mandibular plane angle, an increase in mandibular body length, and an increase in anterior facial height. There were no statistically significant differences observed in other indicators (*P* > 0.05).

**Table 3:** Comparisons of craniofacial morphological variables among the BN, ADD groups

Variables	BN Group1	ADD Group2	*P*	Pairwise comparison
OP-FH	6.579 ± 4.159	7.752 ± 4.055	NS	
AOP-FH	9.028 ± 3.649	10.541 ± 5.373	NS	
POP-FH	13.707 ± 3.933	17.206 ± 4.949	0.001	1 < 2
SN-OP	15.790 ± 4.405	18.064 ± 4.988	0.032	1 < 2
MP-OP	15.552 ± 4.057	17.020 ± 5.124	NS	
PP-OP	5.721 ± 3.249	7.580 ± 4.018	0.03	1 < 2
SNA	82.838 ± 3.315	80.761 ± 3.841	0.013	1 > 2
SNB	80.186 ± 2.988	76.620 ± 3.901	< 0.001	1 > 2
ANB	2.648 ± 1.923	4.142 ± 2.230	0.002	1 < 2
FMA	22.048 ± 5.142	24.770 ± 5.713	NS	
MP-SN	30.997 ± 5.320	35.084 ± 6.453	0.003	1 < 2
Ar-Go-Me	117.172 ± 5.997	117.880 ± 6.411	NS	
PP-MP	21.062 ± 4.714	24.504 ± 6.438	0.004	1 < 2
N-S-Ar	123.024 ± 5.950	127.928 ± 5.765	0.001	1 < 2
Ar-Go	49.969 ± 4.805	51.870 ± 6.113	NS	
Go-Me	70.021 ± 4.704	75.038 ± 5.238	< 0.001	1 < 2
Co-Gn	111.434 ± 7.949	117.987 ± 8.037	0.001	1 < 2
N-Me	116.772 ± 8.292	127.472 ± 8.819	< 0.001	1 < 2
S-Go	80.807 ± 6.990	80.903 ± 7.188	NS	
Condyle height	22.276 ± 1.945	23.065 ± 2.971	NS	

NS not significant

### Comparison of occlusal plane and craniomaxillofacial morphological features among BN, ADDWR and addwor groups

**Fig. 6:**
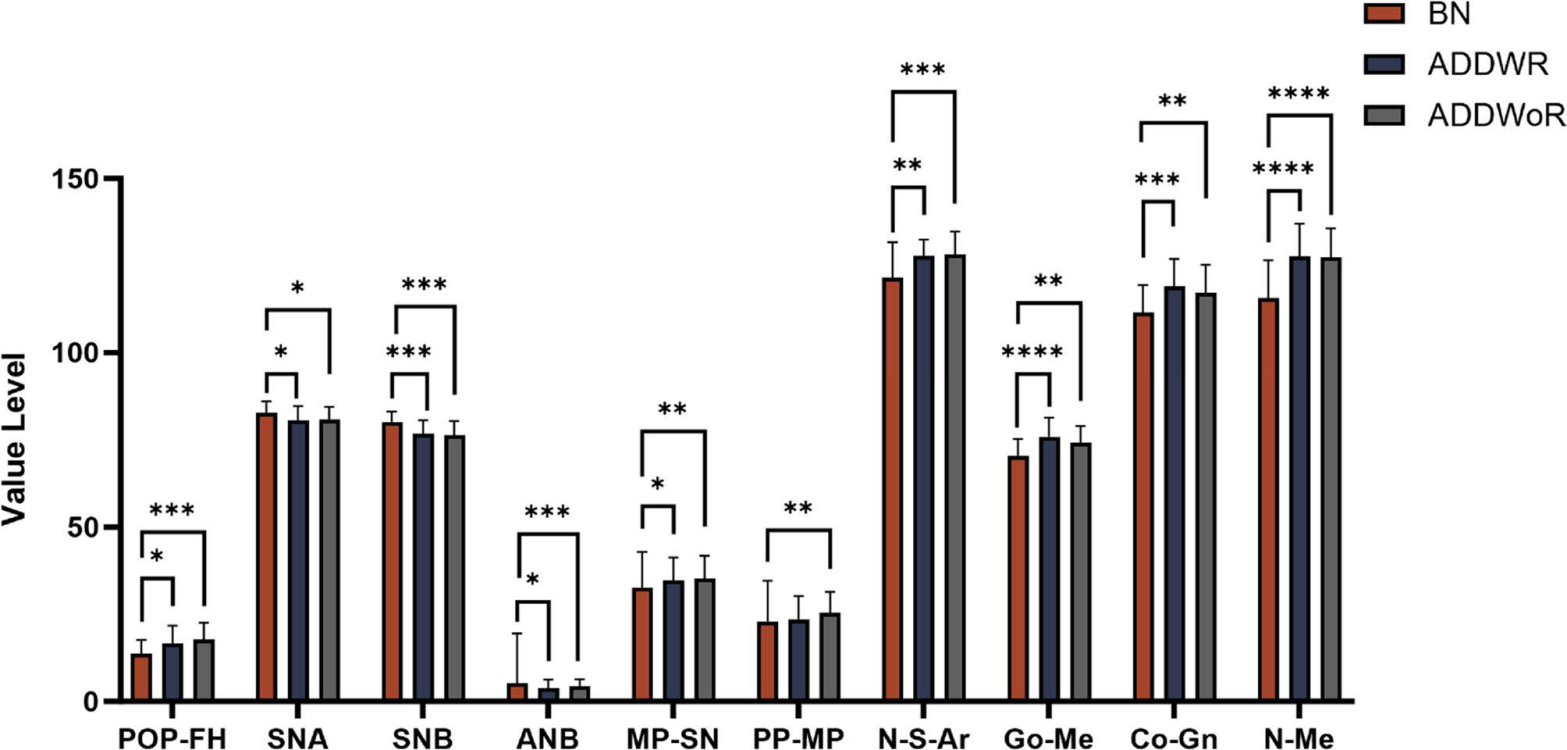
Significantly different measurements of craniomaxillofacial morphology between BN, ADDWR and ADDWoR group. NS not significant; **p ≤ 0.01, ***p ≤ 0.001, ****p ≤ 0.0001 Multiple comparisons were used to analyze the intergroup difference at the level of α = 0.05

The results of a one-way ANOVA and pairwise comparisons conducted among the BN, ADDWR, and ADDWoR groups are as follows. (Table 4; Fig. 6) The previously found statistical differences in SN-OP, PP-OP were not maintained among the three groups. However, the other indicators that had previously shown changes remained exhibited statistically significant differences among the BN, ADDWR, and ADDWoR groups. The data demonstrate that there is a significant measurement decline in SNA and SNB (*P* < 0.01) when comparing BN with ADDWR groups, while POP-FH(*P* < 0.05), ANB, MP-SN, PP-MP, N-S-Ar, Go-Me, and N-Me show an increase. However, comparisons between ADDWR and ADDWoR groups revealed no statistically significant differences in any of these indicators.

**Table 4:** Comparisons of sagittal variables among the BN, ADDWR and addwor groups

Variables	BN Group1	ADDWR Group2	ADDWoR Group3	*P*	Multiple Comparison
OP-FH	6.579 ± 4.159	8.127 ± 4.318	7.405 ± 4.100	NS	
AOP-FH	9.028 ± 3.649	10.558 ± 4.811	10.093 ± 4.956	NS	
POP-FH	13.707 ± 3.933	16.685 ± 5.043	17.683 ± 4.884	0.003	1 < 2 = 3
SN-OP	15.790 ± 4.405	18.361 ± 5.029	17.792 ± 5.007	NS	
MP-OP	15.552 ± 4.057	16.306 ± 4.656	17.492 ± 5.489	NS	
PP-OP	5.721 ± 3.249	7.070 ± 4.573	8.047 ± 3.432	NS	
SNA	82.838 ± 3.315	80.612 ± 4.157	80.897 ± 3.581	0.043	1 < 2 = 3
SNB	80.186 ± 2.988	76.809 ± 3.789	76.447 ± 4.047	< 0.001	1 < 2 = 3
ANB	2.648 ± 1.927	3.803 ± 2.488	4.453 ± 1.949	0.004	1 < 2 = 3
FMA	22.048 ± 5.142	24.633 ± 6.320	24.894 ± 5.183	NS	
MP-SN	30.997 ± 5.320	34.861 ± 6.473	35.289 ± 6.520	0.013	1 < 2 = 3
Ar-Go-Me	117.172 ± 5.997	118.006 ± 5.974	117.764 ± 6.871	NS	
PP-MP	21.062 ± 4.714	23.473 ± 6.827	25.450 ± 6.000	0.016	1 < 3
N-S-Ar	123.024 ± 5.960	127.758 ± 4.741	128.085 ± 6.631	0.001	1 < 2 = 3
Ar-Go	49.969 ± 4.805	51.918 ± 5.883	51.825 ± 6.399	NS	
Go-Me	70.021 ± 4.704	75.967 ± 5.507	74.186 ± 4.900	< 0.001	1 < 2 = 3
Co-Gn	111.435 ± 7.949	118.924 ± 7.989	117.128 ± 8.098	0.002	1 < 2 = 3
N-Me	116.772 ± 8.292	127.624 ± 9.460	127.333 ± 8.321	< 0.001	1 < 2 = 3
S-Go	80.807 ± 6.990	81.282 ± 7.774	80.556 ± 6.699	NS	
Condyle height	22.276 ± 1.945	23.230 ± 2.988	22.914 ± 2.990	NS	

NS not significant

### Correlation analysis between occlusal plane and craniomaxillofacial features (Fig. 7)

Our results present a heatmap displaying the correlation analysis between indicators related to the occlusal plane and positive craniomaxillofacial indicators for the three groups combined. SNB, ANB, FMA, MP-SN, and Ar-Go all have statistical correlations with OP-related indicators.

**Fig. 7:**
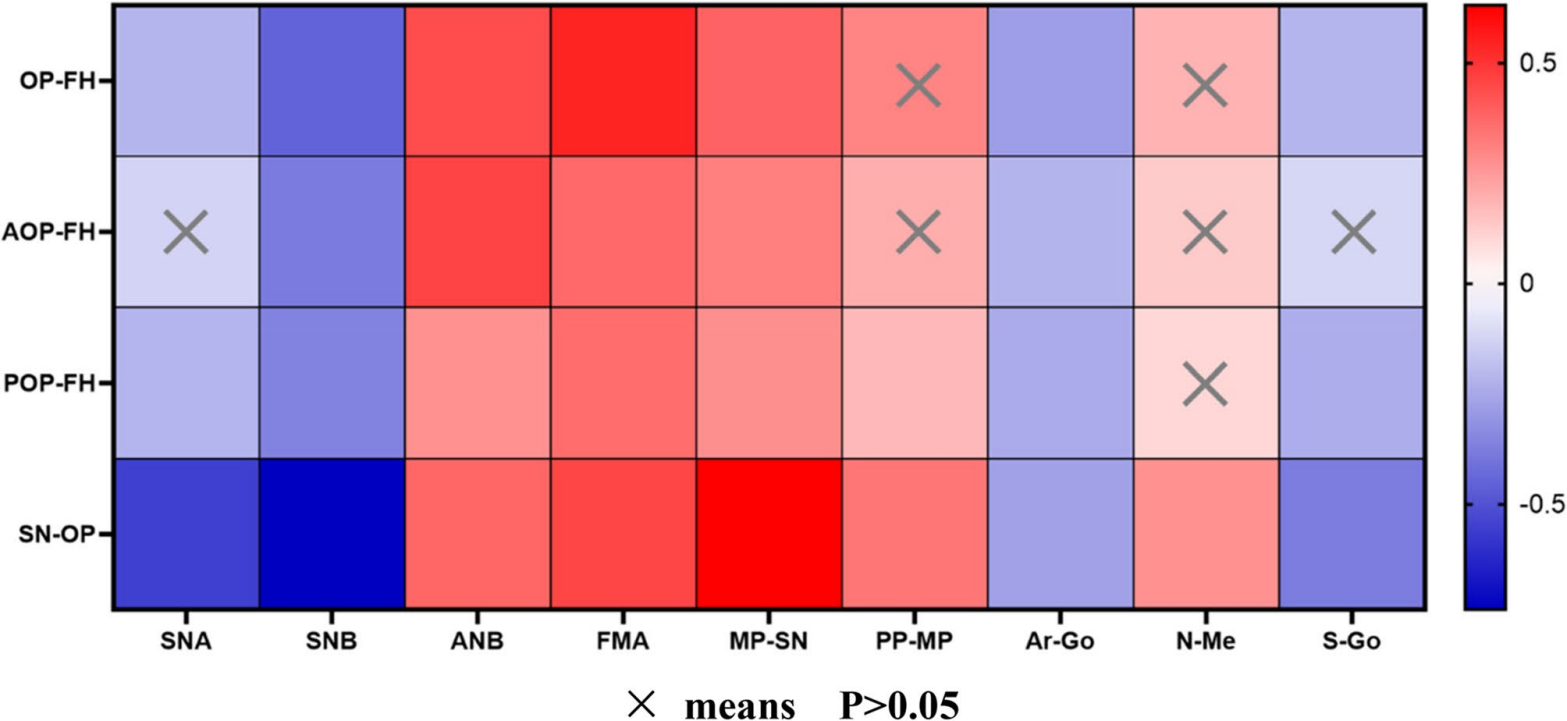
Correlation analysis between OP-related measurements and craniomaxillofacial morphology measurements. **×**means P>0.05

Statistically significant associations (|r| >0.6) were observed between SN-OP and SNB, MP-SN. The correlation (r = -0.739) between SN-OP and SNB suggests a significant positive correlation between a steeper occlusal plane and mandibular retraction.

Moderate correlations (0.4 < |r| < 0.6) were found between OP-FH and SNB, ANB, FMA; AOP-FH and ANB; SN-OP and SNA, FMA. Several other indicators exhibited weak or nonexistent correlations.

## Discussion

Long-term follow-up studies have shown that the peak age for TMD is between 18 and 44 years, and female patients are around 2.24 to 5 times more prone to developing the disease compared to male patients [18]. The severity of degenerative changes in TMD increases with age [19]. Hence, in order to mitigate the impact of age [20], this study limited the age range of participants to 18–44 years, with female subjects being three times more prevalent than male ones.

Before the appearance of RDC/TMD diagnostic criteria, it was difficult for scholars to reach an agreement on the diagnostic and classification criteria of TMD. In 1992, Dworkin et al. Proposed RDC/TMD. Its important significance can be summarized as that RDC/TMD enables clinicians and researchers to use common diagnostic criteria, naming and classification methods for academic research and clinical experience exchange [21]. The validation of the content validity of RDC/TMD Axis I is tested by using epidemiological data through the critical comments of experts, and a multi-center study proves that RDC/TMD shows sufficient reliability for common TMD [22]. The application of RDC/TMD criteria to patient medical histories and clinical examinations yields diagnostic classifications characterized by high specificity, demonstrating enhanced reliability in differential diagnosis [23].

The objective of this study is to examine the morphological alteration in the craniomaxillofacial region of patients with ADD in comparison to those without the disorder, with a particular focus on changes in the occlusal plane (OP). The overall findings suggest that patients with ADD suffer significant craniofacial morphological alterations in comparison to those with bilateral normal disc position (BN). Specifically, there is a clockwise rotation of the OP, an increase in the posterior occlusal plane and mandibular plane angle, a maxillary and mandibular retraction, an increase in the saddle angle and anterior height, and an increase in mandibular body length, suggesting a tendency towards a skeletal Class II high-angle pattern. This indicates a relationship between craniomaxillofacial morphological changes and anterior disc displacement.

### Changes in the occlusal plane

Our research findings demonstrate significant changes in the occlusal plane among patients with ADD. In the ADD group, compared to the BN group, the occlusal plane shows clockwise rotation and the posterior occlusal plane exhibits a steepening trend. Normally, the TMJ disc is positioned between the anterior inclined plane of condyle and the articular tubercle, with the posterior zone of the TMJ disc situated above the transverse ridge. Upon displacement of the TMJ disc, the bilaminar zone advances, resulting in increased tension on the posterior ligament and subsequent posterior and superior movement of the condyle [24]. This motion induces the secretion of cytokines and pertinent enzymes in the bilaminar zone. The surface of the condyle is composed of fibrocartilage, comprises the articular surface layer, proliferative layer, hypertrophic layer, and calcified cartilage layer. The proliferative layer, with stem cell characteristics, serves as the histological foundation for condylar regeneration. However, under the influence of cytokines and enzymes, the proliferative layer may be disrupted, potentially affecting the height of the condyle [25, 26]. Schudy [27, 28] proposed that the growth of the condyle is crucial for alterations in occlusal plane. As the vertical height of the molars surpasses the vertical development of the condyle, the mandible rotates backward, resulting in mandibular retraction. However, Moss and Salentijn [29, 30] argued that facial skeletal growth is primarily influenced by dental function, followed by the growth of the condyle and sutures. Petrovic et al. [31, 32] suggested that occlusal function regulates mandibular growth, enabling the mandible to adapt to the spatial position of the maxillary occlusal plane and dental arch by means of growth or repositioning of the TMJ. Following a 14-year follow-up of 25 children, Kim et al. [11] found that the inclination of the posterior plane, particularly the posterior plane, and the vertical height of the posterior teeth may significantly influence the development of various sagittal bone planes. In the skeletal class II population, the mandibular first molar erupts excessively during the peak growth and development period, resulting in a steepening of the corresponding posterior plane.

Our investigation found no significant change in condylar height in the ADD group (Table 3), possibly due to dental function. The vertical height increase of the posterior teeth exceeds the growth of the condyle, correlating with a steeper posterior plane and a vertical growth pattern, which subsequently observed in conjunction with TMJ disc displacement. Nonetheless, the determinants influencing the orientation of the posterior plane and the precise process of ADD require additional investigation.

Meanwhile, owing to the inherent limitations of cross-sectional studies, it remains unclear whether patients with ADD are more prone to changes in the occlusal plane or whether alterations in the occlusal plane contribute to the occurrence of ADD. Prospective cohort studies are needed to further elucidate this causal relationship. A finite element analysis study [33] indicate that OP inclination changed the differential pattern of force applied on the TMJ disc both in direction and magnitude. Although the etiology of temporomandibular disorders has not yet been established, increased joint loads are reported to be a possible reason for some temporomandibular disorders [34].

### Craniofacial morphological changes

Based on our measurements, patients who have experienced ADD demonstrate maxillary and mandibular retraction, an increase of mandibular plane angle, saddle angle, mandibular body length, effective mandibular length and anterior facial height, presenting a skeletal Class II high-angle mandibular retraction profile tendency. These findings are in line with previous research [35–38]. Previous studies have frequently reported a reduction in mandibular ramus height. However, in our study, the length of the mandibular body and the effective mandibular length were seen to increase in the ADD group. Differences in ethnicity and sample selection​​ may be one of the reasons for the discrepancy in the results. The alterations in dental function resulting from changes in the posterior plane may exert a more significant influence than condylar remodeling and could serve as a critical determinant. However, Jei-jun Shi et al. [39] found no statistically significant variation in the height of the mandibular ramus between the two groups, leading them to suggest that sagittal development of the mandible may be related to certain TMJ disc displacement, whereas vertical changes require further refinement in experimental design and methodology. A study [40] indicates that during mandibular growth, mice fed soft diets exhibit decreased bone mass in the condyle and masseter attachment regions compared to those fed hard diets. Additionally, another study [41] on unilateral chewing show that it might cause muscle imbalances on both sides and affect normal growth and development of the mandible. These studies suggest that strength of the masticatory muscles may impact the growth of the mandible.

Moreover, differences in mandibular morphology between the Uyghur and Han populations have been noted [42]. In our study, patients in the ADD group exhibited increased mandibular body length. Greater masticatory muscle strength throughout growth may be responsible for the larger mandibular body length observed in ADD patients. Our hypothesis is that the length of the mandibular body may be influenced by masticatory muscle strength, ethnic variations, sample size. The influence of changes in the posterior plane on the function of the dental arch may surpass that of condylar remodeling and could be one of the critical factors. There might also be other undiscovered variables. Currently, the vertical changes in craniofacial morphology in patients with anterior disc displacement remain controversial, requiring the implementation of more rigorous research methodologies.

Using MRI, Gökalp et al. [43] investigated TMJ disc displacement in various malocclusions in adolescents, finding a significantly higher prevalence of TMJ disc displacement in skeletal Class II malocclusions than in other forms. This might be attributed to the displacement of TMJ disc and resorption of condylar bone causing posterior-inferior rotation of the mandible. The rotation increases the angle of the mandibular plane and leads to the formation of secondary skeletal Class II facial pattern. Therefore, orthodontists should highly suspect TMJ disc displacement in skeletal Class II patients and recommend MRI or CBCT imaging to accurately assess joint condition for optimal treatment planning.

Furthermore, in this study, the SNA and SNB angles of the ADD group were both diminished compared to the normal group, while the saddle angle exhibited an increase. Previous research consistently demonstrated a reduction in the SNB angle; however, findings on the SNA angle varied [44–46]. A longitudinal study with a follow-up time of over three years by Carlos Flores-Mir et al. pointed out that TMJ disc abnormality was associated with reduced forward growth of the maxillary and mandibular bodies, which supported the results in our study [47]. Järvinen [48] found that among children in Angle Class I, the notable alteration in SNA angle can be attributed to the modification in saddle angle, demonstrating an inverse correlation between the two. Upon retraction of the maxilla, a smaller SNA angle corresponds to a bigger saddle angle, signifying that the lower jaw is adequately retracted, which reflects a harmonious growth and development between the upper and lower jaws. Another study [49] backs up this finding.

In intergroup variance analysis, the dentofacial changes associated with ADD were greater between subjects with BN and ADDWR than between those with ADDWR and ADDWoR.

Despite patients in the ADDWoR group exhibiting more severe TMJ disc lesions, their associated indicators, such as the posterior plane, did not deteriorate further, suggesting that craniofacial morphological alterations were more pronounced in the initial stages of anterior disc displacement, while previous studies have shown different results [44, 46, 50].

Studies [35,36,37,38,51] show that as the degree of TMJ disc displacement advances from BN to ADDWR to ADDWoR, skeletal Class II features become increasingly pronounced. This may be attributed to progressive changes in condylar morphology, associating with a more severe skeletal Class II profile. In our study, from ADDWR to ADDWoR, no statistically significant differences were observed in occlusal plane or sagittal and vertical craniofacial indicators, suggesting that changes in condylar morphology alone may cannot explain these findings. Petrovic’s theory [31, 32] posits that occlusal function regulates growth of the mandible, enabling it to adapt to the maxillary occlusal plane and dental arch’s spatial position through TMJ growth or repositioning. Moss et al. [29, 30] suggested that condylar cartilage undergoes growth in response to changes in the mandibular position, and that the position of the teeth affects face growth. Ye [52] found a notable correlation between inclination of the posterior occlusal plane and sagittal mandibular position. Specifically, the steepness of the posterior occlusal plane increases when the mandibular retraction occurs. As the posterior occlusal plane flattens during growth, the angle of the mandibular plane decreases, causing the mandibular position to advance [53]. Our intergroup variance analysis showed no differences in the posterior occlusal plane between the ADDWR and ADDWoR groups. Correspondingly, there were no statistical differences in SNA, SNB, Go-Me, and N-Me, suggesting that changes in the posterior occlusal plane influence sagittal and vertical craniofacial indicators. The MP-SN and ANB did not show statistical differences but showed a gradual increase in mean values, possibly due to the small sample size and non-normal distribution of ANB values in the TMJ disc displacement groups.

### Correlation analysis

In our study, SNA, ANB, FMA, MP-SN and Ar-Go were correlated with all OP-related indicators. And most OP -related indicators had correlation with SNA and S-Go, but less with PP-MP and N-Me. Vertically, FMA and MP-SN are frequently employed for the analysis and evaluation of mandibular plane angles [54]. Both variables had positive correlations with OP indicators in this study, and SN-OP exhibits a high correlation with MP-SN, suggesting that an excessive extent of vertical growth was associated with a steeper OP, albeit with a somewhat moderate link. An increase in the FMA angle and MP-SN angle associate witha clockwise rotation of the mandible, known as mandibular retraction. Previous studies have found that a gradual flattening of the maxillary OP leads to a reduction in the FH-MP angle (FMA) [6], consistent with our findings. The findings of our study demonstrate that vertical indicators can partially reflect the tendency for OP in clinical settings. Maxillary indicator, namely SNA exhibited a less pronounced correlation with OP. In the mandibular indicators, SNB and Ar Go are negatively correlated with OP indicators, with a strong correlation between SNB and SN-OP indicating that the smaller the SNB angle, the lower the mandibular ramus height, that is, the more posterior the mandible is in relation to the craniofacial area, and the larger the OP angle, which may be associated with mandibular rotation during dental arch construction. These findings suggest that both the anterior and posterior occlusal planes may can influence the direction of mandibular rotation and its sagittal and vertical positions, ultimately affecting facial aesthetics and function.

Although the present study demonstrates a strong association among TMJ disc displacement, occlusal plane, and craniomaxillofacial morphology, the precise causal connection between these factors remains uncertain. Craniomaxillofacial morphological changes can arise from several causes, such as disorders of the TMJ, alterations in the occlusal plane that affect the structures and pressures within the TMJ, or both TMJ disc displacement and occlusal plane changes influenced by unknown factors. Longitudinal studies may help establish the causal relationship and correlation among disc displacement, occlusal plane, and sagittal craniofacial morphology.

Additionally, the posterior rotation of mandible, backward position of mandible, dolichofacial (hyperdivergent) profile, increased posterior occlusal plane etc. are directly correlated with orthopedic instability (large CR-MI discrepancy). Studies [55, 56] demonstrated that such discrepancies create a musculoskeletal imbalance, where occlusal interferences force the mandible into a compensatory posture, displacing condyles from their stable position in the fossa. These adaptations imperceptibly overload the lateral pterygoid and elevator muscles, generating masticatory dysfunction that may contribute to the development of TMDs and orofacial pain. Importantly, establishing an orthopedically stable musculoskeletal position with an occlusal splint therapy in patients with posterior rotation of mandible is helpful, which reduce signs and symptoms of TMDs, provide more harmonious relation between the CR and MI, ensures the leveling of the occlusal plane and adequate starting position for diagnosis and treatment planning of orthodontic cases [57].

Orthodontists in clinical practice must remain attentive to TMD in patients exhibiting skeletal anomalies, particularly those with skeletal Class II profiles. For orthodontic patients experiencing ADD, it is important to use multiple treatment strategies depending on the specific joint problems and craniomaxillofacial structure. Attempts should be made to restore disc-condyle relationships in growing adolescents to promote condylar growth and improve facial profiles. Combined orthodontic, orthognathic, and joint treatments are advisable for adult patients with severe facial abnormalities to improve facial profiles and enhance life quality.

### Limitations

This study has the following four shortcomings. Firstly, RDC/TMD clinical diagnostic criteria provide a standardized process for disc displacement, but its sensitivity and specificity are limited. For example, the diagnosis of anterior disc displacement in this standard mainly depends on the palpation method with strong subjectivity. It is recommended that follow-up studies include MRI detection to avoid the deviation of clinical decision-making caused by excessive reliance on subjective indicators. Secondly, we didn’t include other contributing factors, such as large CR-MI discrepancy, psychological factors (emotional stress etc.), trauma, loss of posterior teeth, facial asymmetry, bruxism, chewing pattern, impact of unilateral clenching etc. Thirdly, the causal relationship between TMJ disc displacement and occlusal factors is not clear because the results were derived from cross-sectional data, therefore a cohort study should be used to investigate to further investigate the causal relationship. Fourthly, as the disc displacement progresses from ADDWR to ADDWoR, the condylar position varies greatly, and the specific changes need to be further studied by increasing the sample size.

### Suggestions for future studies

In the most of the earlier studies there was not found a specific relation between the orthodontic malocclusions and TMD. Findings related to these etiologic factors can be correlated with orthodontic malocclusion of TMJ disc. If only craniofacial features were considered, as in this study, then individuals with clockwise rotated mandible, increased overjet and shorter ramus height are more predisposed for TMD. On the other side, when we consider the patients with anticlockwise rotation of mandible (brachycephalic), they usually grind and clench their teeth more and are also predisposed to development of TMD. This is very individual and depends on numerous factors, take into account patient’s adaptability capacity. For this reason, further studies should correlate results with all other etiologic factors that are pertained to a certain individual. On the basis of DC/TMD protocol and analysis of occlusion using condylar position indicator (CPI), patient’s occlusion (presence or non-presence of orthopedic instability), emotional factors, parafunctional activities (grinding, clenching), presence of deep somatic pain, possible trauma etc. should be individually determined and correlated with the craniofacial morphology. In order to be relevant, it is strongly suggested to include more elements in further studies. And, to minimize potential confounding, ​​future studies should select​​ control groups (BN) with different vertical or sagittal skeletal facial patterns, such as Skeletal Class I subjects with normodivergent facial patterns. Finite element analysis study may provide more reliable data regarding the association between craniomaxillofacial morphological changes and ADD.

## Conclusion

1. ADD patients have an increase of the occlusal plane, especially the posterior occlusal plane.
2. The craniomaxillofacial morphology in patients with ADD ​​was associated with​​ a backward position of the mandible and an increase of the mandibular plane angle.
3. Significant dentofacial changes were found mainly between BN and ADDWR, indicating that dentofacial morphologies may begin to change during the initial stage of TMJ ADD.
4. There is a notable association between the occlusal plane and the skeletal Class II high angle trend in the population.

## Data Availability

No datasets were generated or analysed during the current study.

## Abbreviations

BN: Bilateral Disc Normal Position
ADDWR: Anterior disc displacement with reduction
ADDWoR: Anterior Disc Displacement Without Reduction
TMJ: Temporomandibular joint
TMD: Temporomandibular disorders
ID: Internal disorders
OP: Occlusal plane
AOP: Anterior occlusal plane
POP: Posterior occlusal plane
RDC/TMD: Research Diagnostic Criteria for Temporomandibular Disorder
CBCT: Cone-beam computed tomography
MRI: Magnetic Resonance Imaging
ANOVA: Analysis of variance
